# Malignant schwannoma of the upper mediastinum originating from the vagus nerve

**DOI:** 10.1186/1477-7819-3-65

**Published:** 2005-10-06

**Authors:** Fumihiro Shoji, Riichiroh Maruyama, Tatsuro Okamoto, Hiroshi Wataya, Kenichi Nishiyama, Yukito Ichinose

**Affiliations:** 1 Department of Thoracic Oncology, Kyushu Cancer Center, 3-1-1, Notame, Minami-ku, Fukuoka 811-1395, Japan; 2 Department of Pathology, Kyushu Cancer Center, 3-1-1, Notame, Minami-ku, Fukuoka 811-1395, Japan

## Abstract

**Background:**

Malignant schwannoma of the upper mediastinum originating from the vagus nerve is extremely rare.

**Case presentation:**

A 46-year-old female was admitted for a left cervical mass which was associated with both hoarseness and Horner's syndrome. Chest computed tomography showed a mass extending from the left upper mediastinum to the left supraclavicular area. A fine needle aspiration cytological examination suggested primary lung cancer stage IIIB large cell carcinoma. After administering induction chemo-radiotherapy, a complete surgical resection was performed. The tumor was found to involve both the left vagus nerve and the left sympathetic nerve. Histological examination of the resected specimen revealed the tumor to be malignant schwannoma.

**Conclusion:**

Despite incorrect preoperative diagnosis, the multimodality treatment administered in this case, including induction chemo-radiotherapy and surgery, proved to be effective.

## Background

According to a collected series of 2399 cases of mediastinal tumors reported in the literature [1], 496 cases (20.7%) were of neurogenic tumors, and most of them occurred in the posterior mediastinum. Neurogenic tumors can be divided into two groups depending on their origin: those that arise from the nerve sheath and those that arise from nerve cells. The majority of the tumors of nerve sheath origin in adults are either benign schwannomas or neurofibromas, and they usually arise from either an intercostal nerve or a sympathetic nerve. Intrathoracic schwannoma originating from the vagus nerve, is extremely rare.

## Case presentation

**Figure 1:**
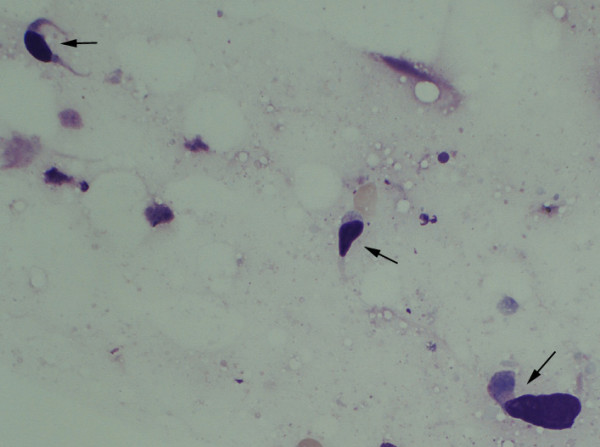
Aspiration cytology of the tumor before treatment showing scattered atypical spindle cells (arrows) (Giemsa staining, high power view x400).

**Figure 2:**
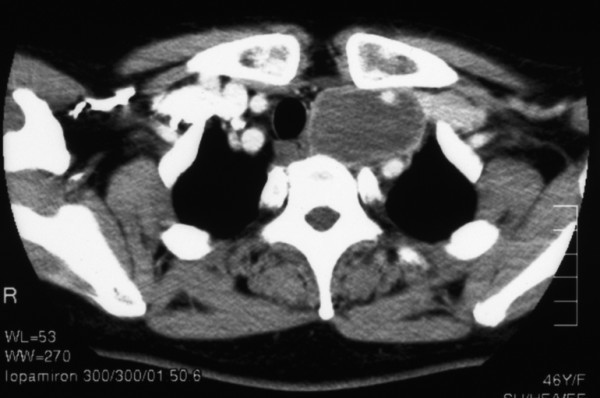
Chest CT showed a mass, measuring 5.0 cm in size in the upper mediastinum.

**Figure 3:**
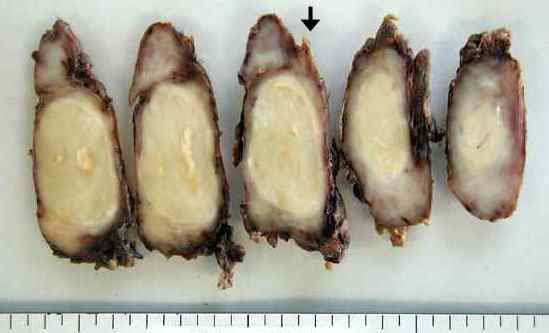
Macroscopic findings of the tumor. The tumor showing the continuity of the vagus nerve (arrow head) was whitish in color and oval shaped while measuring 5 × 3 cm in diameter.

**Figure 4:**
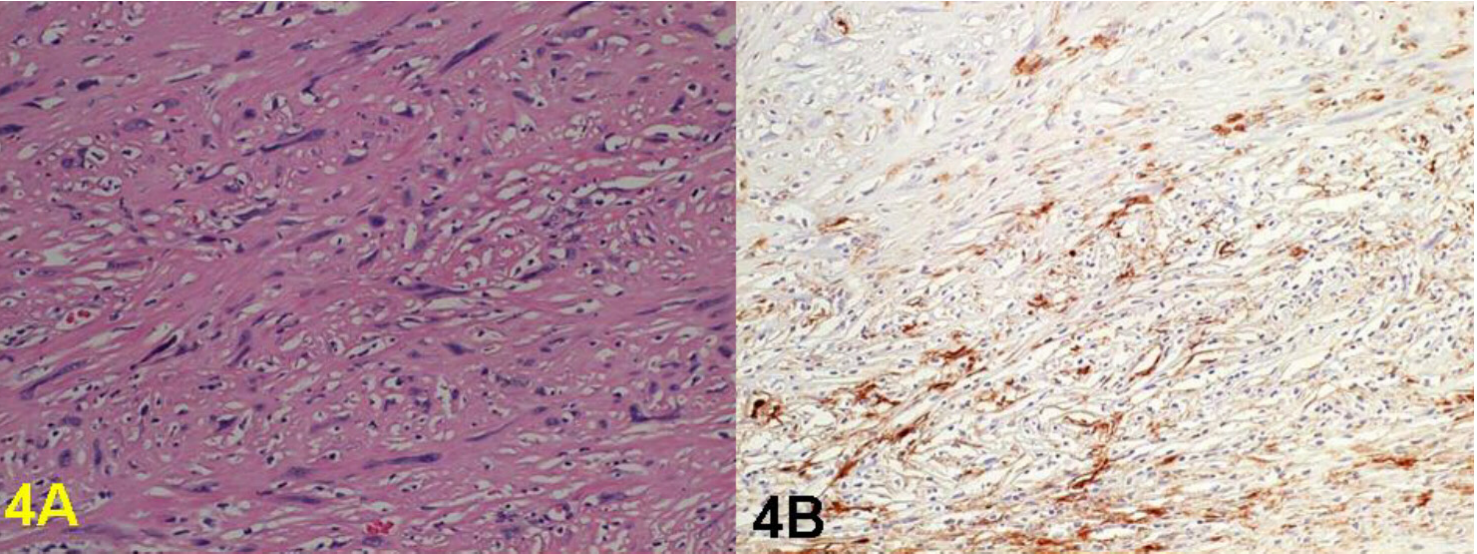
Histological findings of the tumor. 4A) The tumor was found to consist of a few of viable atypical spindle cells with hyperchromatic nuclei (Hematoxilin-eosin staining x200) 4B). Tumor cells were positive for S-100 protein (immunohistochemical staining, original magnification: X200).

A 46-year-old female with symptoms of hoarseness and Horner's syndrome presented with a left cervical mass that was diagnosed to be undifferentiated carcinoma based on the findings of aspiration cytology (Figure 1). The patient's chest computed tomography (CT) findings showed a mass measuring 5.0 cm in size spreading from the left upper mediastinum to the left supraclavicular area, which pressed against both the trachea and esophagus and it seemed to involve the left common carotid artery (Figure 2). Based on these findings, and cytology findings a clinical diagnosis of stage IIIB (T4N3M0) non-small cell lung cancer (NSCLC) originating from the apex of the left lung involving both the mediastinum and the supraclavicular lymph nodes was made [2]. The patient received concurrent chemo-radiotherapy (cisplatin 80 mg/m^2 ^for days 8 and 36 + UFT 400 mg/m^2^, both on days 1–14 and on days 29–42 plus radiotherapy, 2 Gy/day on days 1–20 for a total of 40 Gy) [3]. After this treatment regimen, the tumor size decreased by 35.0%. Thereafter, the patient underwent a surgical resection though a median sternotomy with a combined resection of the left clavicle. During the operation, an encapsulated tumor was detected in the mediastinum. Although the tumor was easily ablated from the left common carotid artery, it involved both the left vagus and the sympathetic nerves. As a result, both nerves had to be sacrificed in order to achieve a complete resection of the tumor. Grossly, the tumor was in continuity of the vagus nerve was whitish in color and oval shaped measuring 5 × 3 cm in diameter (Figure 3). Both cytological and histological examinations revealed 1) Continuity between the vagus nerve and tumor was seen, while the no continuity between the tumor and the sympathetic nerve was found. 2) The findings of aspiration cytology of the tumor diagnosing it to be undifferentiated carcinoma before the treatment included an atypical spindle cell. (Figure 1). 3) The predominantly tumor consisted of necrotic tissue and a few viable atypical spindle cells (Figure 4A) which were positive for S-100 protein (Figure 4B). As a result, the tumor was considered to arise from the left vagus nerve while invading the left sympathetic ganglion, and was therefore diagnosed it to be a malignant schwannoma. At present, the patient has survived for about 2 years since operation without any recurrence.

## Discussion

Tumors of vagus nerve origin are observed in about 2% of all neurogenic tumors of the mediastinum [4], however, no instace of malignant schwannoma was reported in this review. To our knowledge, only a few such cases have been previously reported [5-7]. As a result, malignant schwannoma originating from the vagus nerve is therefore considered to be extremely rare.

Malignant peripheral nerve sheath tumors (MPNST) including malignant schwannoma are the malignant variants of schwannomas and neurofibromas. Although the 5-year survival rates have been reported to be up to 75 % in MPNST's patients, MPNST often advance locally and can also occasionally metastasize to the lung or other organs [8]. Therefore, in addition to a complete surgical resection, adjuvant therapy is usually advocated. However, in an adjuvant setting, the efficacy of chemotherapy or radiotherapy appears to provide little additional benefit [9,10]. We previously reported concurrent chemo-radiotherapy with UFT plus cisplatin as an induction treatment followed by a surgical resection for patients with marginally resectable stage IIIB NSCLC to be both a feasible and promising treatment [3]. Since we preoperatively considered the disease to be marginally resectable stage IIIB NSCLC, we performed concurrent induction chemo-radiotherapy followed by surgery. As a result, this multimodality treatment proved to be effective and the patient is now doing well without any recurrence.

We initially misdiagnosed this patient's disease to be non-small cell lung cancer. The reason for this was partly due to the cytological findings which indicated undifferentiated carcinoma. In general, an exact diagnosis cannot always be made based on the findings of aspiration cytology alone. The second reason for a misdiagnosis in this case was due to the patient's symptoms which included hoarseness and Horner's syndrome. Apical lung cancer involving both the vagus and the sympathetic nerve is occasionally observed. However, to the best of our knowledge, the present case is considered to be the first case demonstrating malignant schwannoma of the vagus nerve involving the sympathetic nerve.

## Conclusion

Malignant schwannoma of the upper mediastinum arising from the vagus nerve is rare. The multimodality treatment administered in this case, including induction chemo-radiotherapy and surgery, proved to be effective.

## Competing interests

The author(s) declare that they have no competing interests.

## Authors' contributions

**FS**: conceived of the study, participated in its design and coordination and drafted the manuscript.

**RM **and **TO**: carried out the literature search and helped in drafting the manuscript.

**HW**: participated in the design of the study and helped in drafting the manuscript.

**KN**: performed histological examination and provided photographs.

**YI**: shaped the idea for the study, coordinated the study and edited the manuscript.

All authors read and approved the final manuscript.
